# Comprehensive analysis of aberrantly expressed profiles of lncRNAs and miRNAs with associated ceRNA network in muscle-invasive bladder cancer

**DOI:** 10.18632/oncotarget.13363

**Published:** 2016-11-15

**Authors:** Hanbo Wang, Leilei Niu, Shaobo Jiang, Jing Zhai, Ping Wang, Feng Kong, Xunbo Jin

**Affiliations:** ^1^ Minimally Invasive Urology Center, Shandong Provincial Hospital affiliated to Shandong University, Jinan, China; ^2^ Department of Surgery, The Chinese University of Hong Kong, Prince of Wales Hospital, Sha Tin, New Territories, Hong Kong SAR, China; ^3^ Department of Biochemistry, School of Basic Medical Sciences, Taishan Medical University, Taian, China; ^4^ Department of Surgery, Qilu Hospital, Shandong University, Jinan, China; ^5^ Central Laboratory, The Second Hospital of Shandong University, Jinan, China

**Keywords:** muscle-invasive bladder cancer, lncRNA, ceRNA, miRNA, TCGA

## Abstract

Although initially thought to be transcriptional noise, long noncoding RNAs (lncRNAs) are gaining increased attention in human cancers as its diversity function. At present, lncRNAs are regarded as the main part of competing endogenous RNA (ceRNA) network due to its regulation on protein-coding gene expression by acting as miRNA sponges. However, functional roles of lncRNA-mediated ceRNAs in muscle-invasive bladder cancer remain unclear. To clarify relevant potential mechanisms, here we comprehensively compared the expression profiles of mRNAs, lncRNAs and miRNAs between 322 muscle-invasive bladder cancer tissues and 19 non-tumor bladder tissues, based on the Cancer Genome Atlas (TCGA). A total of 22 lncRNAs were identified as aberrantly expressed and had correlations with tumorigenesis and/or progression of muscle-invasive bladder cancer (|log2FoldChange| > 1.5, corrected *P value* < 0.01). 6 out of the 22 dysregulated lncRNAs functioned as prognostic biomarkers for patients with muscle-invasive bladder cancer according to the overall survival analysis (*P value* < 0.05). Finally, a dysregulated lncRNA-associated ceRNA network was successfully constructed, which inculdes five muscle-invasive bladder cancer-specific lncRNAs, nine miRNAs and 32 mRNAs. In summary, our study identified novel lncRNAs as candidate prognostic biomarkers and potential therapeutic targets for muscle-invasive bladder cancer, based on large-scale sample size. More importantly, the newly identified ceRNA network will be beneficial for improving the understanding of lncRNA-mediated ceRNA regulatory mechanisms in the pathogenesis of muscle-invasive bladder cancer.

## INTRODUCTION

Worldwide, bladder cancer in males is the second most frequently diagnosed cancer, with estimated 429,800 new cases and 165,100 deaths [1]. Emerging data reveal that bladder cancer possesses a variety of genetic and phenotypic characteristics. Even though patients received systematic therapy, the majority of disease eventually recurs or develops metastases. It has been widely accepted that bladder cancer is a heterogeneous disease with two distinct subtypes: non-muscle-invasive bladder cancer and muscle-invasive bladder cancer. Muscle-invasive bladder cancer is regarded as the major contributor to bladder cancer-related mortality [2]. However, its mechanisms remain largely unknown. In this case, identification of potential biomarkers or therapeutic targets to combat bladder cancer, especially muscle-invasive bladder cancer, is urgently needed.

Long non-coding RNAs (lncRNAs) are non-protein coding transcripts longer than 200 nucleotides [3]. Initially, lncRNAs were regarded as “junk DNA” because they lack a protein coding function. Recently, lncRNA biology in cancers is rapidly gaining attention because accumulating studies demonstrated its important roles in both carcinogenesis and cancer metastasis [4]. However, how lncRNAs act to regulate gene expression has remained enigmatic. Although theoretical and experimental studies have partially revealed that lncRNAs regulate gene expression at different levels including transcription, post-transcription and translation, the complete spectrum of mechanisms of through which lncRNAs affect cancer biology is not clear.

Currently, much effort is being made to elucidate how lncRNAs exert their diversity biological functions in human cancer. The influence of lncRNAs upon microRNA (miRNA) function is rapidly emerging. MiRNAs, another class of non-coding RNAs that have been extensively studied, repress target gene expression through their partial complementarity with the mRNA sequence, which named microRNA response elements (MREs) [5]. It has been well-documented that one miRNA can prevent up to hundreds of mRNA transcripts, while each transcript may be targeted by several different miRNAs. The regulatory networks between miRNAs and target genes involve a variety of biological processes, including carcinogenesis and tumor metastasis [6, 7]. In 2011, salmena and colleagues proposed a competing endogenous RNA (ceRNA) hypothesis that lncRNAs, mRNAs and other RNAs act as natural miRNA sponges to suppress miRNA function by using shared MREs [8]. This hypothesis has been validated experimentally by some researchers that lncRNA functioned as ceRNA to communicate with mRNAs through competing for shared miRNAs [9]. LncRNAs that harbor similar sequence to their targeted miRNAs, can sequester miRNAs away from mRNAs. For example, H19 can affect the expression of endogenous let-7 targets through acting as a molecular sponge to sequester let-7 [10]. lncRNA-ATB up-regulated ZEB1 and ZEB2 by competitively binding to the miR-200 family and then induced EMT [11]. We believe exploration of such a RNA interaction would furnish new clues for cancer therapy. Based on above theory, lncRNA-miRNA-mRNA ceRNA network has been constructed in gastric cancer, hepatocellular cancer, breast cancer and pancreatic cancer [12–15]. However, similar studies are rare in bladder cancer. Furthermore, comprehensive analysis of muscle-invasive bladder cancer-associated lncRNAs and miRNAs in a whole genome wide, especially based on high through detection with large-scale sample size, has always been lacking.

The publically available database, Cancer Genome Atlas (TCGA), which collects approximately 10,000 patient samples together with clinicopathological information across more than 30 human cancer types, is a rich resource for data mining and biological discovery. In our study, we initially analyzed RNA expression profiles between the 322 tumor tissues and 19 non-tumor tissues of muscle-invasive bladder cancer. As a result, 1385 aberrantly expressed RNAs were identified. Seven down-regulated and four up-regulated RNAs were classified as lncRNAs. In order to elaborate the valid potential crosstalk between lncRNA, miRNA and mRNA, we further identified differentially expressed miRNAs between muscle-invasive bladder cancer patients and normal samples. Eventually, five dysregulated lncRNAs, nine miRNAs and 32 mRNAs were selected to construct the lncRNA-miRNA-mRNA ceRNA network, based on bioinformatics prediction and correlation analysis.

## RESULTS

### Patient characteristics

The detailed clinical and pathological characteristics of study population were summarized in Table 1. All 322 patients were pathologically diagnosed as muscle-invasive bladder cancer. The median age for all patients was 69 years (IQR: 60–76 years). Consistent with previous report [16], an obvious male predominance was observed (male to female ratio: 2.97). The majority of patients were the white race (81.7%) and high histologic grade (93.5%).

**Table 1:** Clinicopathological characteristics of 322 patients with muscle-invasive bladder cancer

Parameter	Subtype	Patients *n* (%)
Age (years)	> 68	146 (45.3)
	≤ 68	176 (54.7)
Gender	Male	241 (74.8)
	Female	81 (25.2)
Race	Asian	41 (12.7)
	White	263 (81.7)
	Black or African American	18 (5.6)
Smoking history	Lifelong Non-smoker	88 (27.3)
	Current smoker	68 (21.1)
	Current reformed smoker for < or = 15 years	52 (16.1)
	Current reformed smoker for > 15 years	104 (32.3)
	Not available	10 (3.1)
Tumor status	Tumor free	185 (57.5)
	With tumor	103 (32.0)
	Not available	34 (10.5)
Histologic grade	High	301 (93.5)
	Low	21 (6.5)
Histologic subtype	Non-papillary	218 (67.7)
	Papillary	104 (32.3)
Pathologic stage	Stage II	89 (27.6)
	Stage III	118 (36.6)
	Stage IV	115 (35.7)
Pathologic T	T1	1 (0.3)
	T2	102 (31.6)
	T3	165 (51.2)
	T4	54 (16.8)
Pathologic N	N0	187 (58.1)
	N1	3(11.5)
	N2	6(21.4)
	N3	6 (1.9)
	NX^a^	23 (7.1)
Pathologic M	M0	153 (47.5)
	M1	6 (1.9)
	MX^b^	163 (50.6)

a: regional lymph node unknown; b: metastasis status unknown.

### Differentially expressed RNAs (DERNAs) in muscle-invasive bladder cancer

**Figure 1:**
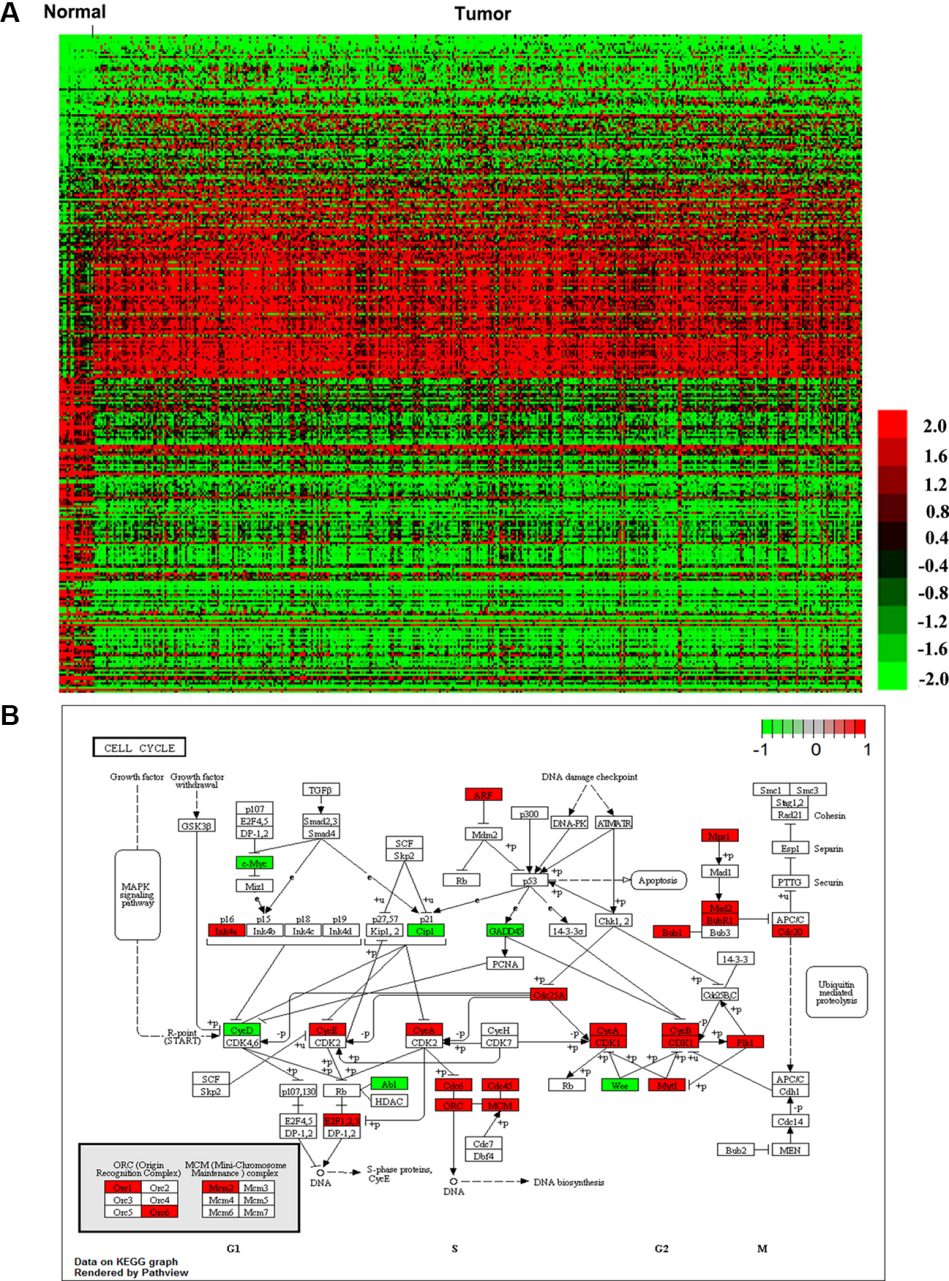
DERNAs in muscle-invasive bladder cancer 1385 DERNAs were identified and relevant pathway analysis using KEGG was performed. (**A**) The top 100 up-regulated and 100 down-regulated DERNAs were visualized by heatmap. Each column represents one sample, and each row indicates a transcript. (**B**) Cell cycle map from KEGG pathway. Up-regulated DERNAs were boxed red, downregulated DERNAs were boxed green.

RNA expression levels in 322 muscle-invasive bladder cancer tumor tissues (Cohort T) and 19 normal tissues (Cohort N) were investigated. Genes with absolute log 2 fold change greater than 1.5 and corrected *P value* less than 0.01 were considered as differentially expressed. As a result, a total of 472 (34.08%) up-regulated and 913 (65.92%) down-regulated genes were identified (Supplementary Table S1). Of those, we outlined top 100 up-regulated and top 100 down-regulated genes in Figure 1A. All of the RNA expression levels were median-centered across samples. The heatmap with complete linkage clustering was carried out by MeV4.9.0. A full list of DERNAs and their corresponding fold changes in expression and *P* values was shown in Supplementary Table S1.

To better understand the mechanisms that involved in the tumorigenesis of muscle-invasive bladder cancer, functional characterization of the DERNAs was performed using KEGG analysis through KOBAS 2.0. A full list of significantly enriched KEGG pathways was provided in Table 2. Cell cycle was found to be the most significant cancer-related pathway (*P* < 0.01) (Figure 1B). As shown in Supplementary Table S2, 27 DERNAs, including 21 up-regulated and 6 down-regulated, were involved in cell cycle. Combined with further analysis, 4 out of the 27 DERNAs, including ABL1, CDKN1A, CDC25A and MYC, had great potential to interact with DElncRNAs and DEmiRNAs (Supplementary Table S2).

**Table 2:** Significantly enriched KEGG pathways regulated by DERNAs in muscle-invasive bladder cancer

Pathway ID	Description	*P*-Value	Corrected *P*-Value	Number of DERNAs
**hsa05410**	Hypertrophic cardiomyopathy (HCM)	1.06E-06	0.000248357	24
**hsa05414**	Dilated cardiomyopathy	3.50E-06	0.000409288	24
**hsa04510**	Focal adhesion	7.83E-06	0.000610513	39
**hsa05412**	Arrhythmogenic right ventricular cardiomyopathy (ARVC)	5.45E-05	0.003190045	19
**hsa04270**	Vascular smooth muscle contraction	9.01E-05	0.004217222	25
**hsa04022**	cGMP-PKG signaling pathway	0.000174	0.006786746	30
**hsa04020**	Calcium signaling pathway	0.000531	0.017754275	30
**hsa04921**	Oxytocin signaling pathway	0.000766	0.021187657	27
**hsa04512**	ECM-receptor interaction	0.000815	0.021187657	18
**hsa04713**	Circadian entrainment	0.001048	0.02453233	19
**hsa04110**	Cell cycle	0.001383	0.029420859	27

Abbreviations: KEGG, Kyoto Encyclopedia of Genes and Genomes.

Statistical test method: hypergeometric test/Fisher's exact test.

FDR correction method: Benjamini and Hochberg.

### Differentially expressed lncRNAs (DElnRNAs) in muscle-invasive bladder cancer

**Figure 2:**
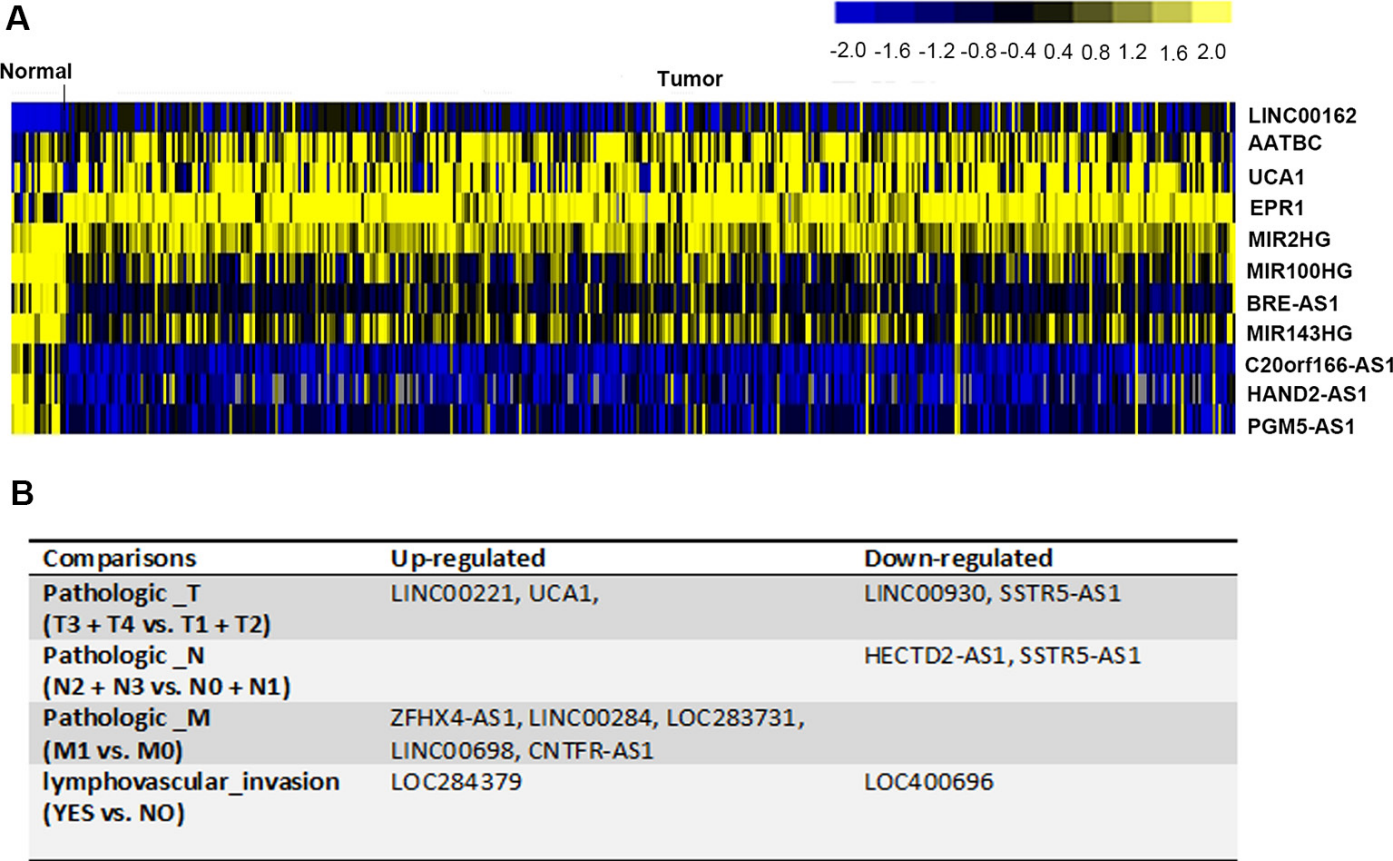
DElncRNAs in muscle-invasive bladder cancer (**A**) The heatmap showed 11 aberrantly expressed lncRNAs in muscle-invasive bladder cancer tissues, compared with normal tissues. They were considered as muscle-invasive bladder cancer initiation-related. (**B**) 12 lncRNAs that associated with the development and progression of muscle-invasive bladder cancer were identified.

According to the cut-off criteria of |log2FoldChange| > 1.5 and corrected *P value* < 0.01, 11 lncRNAs were identified as aberrantly expressed in muscle-invasive bladder cancer tissues compared to the normal tissues (Figure 2A). The expression of four lncRNAs, LINC00162, AATBC, UCA1, and EPR1, was significantly elevated in the malignant tissues. Seven lncRNAs, MIR2HG, MIR100HG, BRE-AS1, MIR143HG, C20orf166-AS1, HAND2-AS1, and PGM5-AS1, were shown to be down-regulated in muscle-invasive bladder cancer (Figure 2A). The data suggest that those 11 lncRNAs were implicated in the tumorigenesis of muscle-invasive bladder cancer.

To further investigate whether lncRNAs participated in the development and progression of muscle-invasive bladder cancer, tumor tissue samples were divided into several subgroups according to pathological tumor-node-metastasis (TNM) stages (T3+T4 vs. T1+T2, N2+N3 vs. N0+N1, M1 vs. M0) as well as with or without lymphovascular invasion (Yes vs. No). Relevant comparison analysis for lncRNA expression profiles was conducted. We identified 8 lncRNAs of high level (LINC00221, UCA1, ZFHX4-AS1, LINC00284, LOC283731, LINC00698, CNTFR-AS1, and LOC284379) and 4 lncRNAs of low level (LINC00390, SSTR5-AS1, HECTD2-AS1, and LOC400696) were significantly related to the progression of muscle-invasive bladder cancer (Figure 2B). UCA1 not only identified as differentially expressed in tumor tissues (Tumor vs. Normal, Figure 2A) but also promoted tumor growth (T3+T4 vs. T1+T2, Figure 2B), suggesting its promotional role in tumorigenesis and growth of muscle-invasive bladder cancer. Another remarkable signature was SSTR5-AS1, which was decreased in samples with high level of pathological node and metastasis stage, implying that SSTR5-AS1 played a negative role during the regional lymph node metastasis and distant organ metastasis of muscle-invasive bladder cancer (Figure 2B).

**Figure 3:**
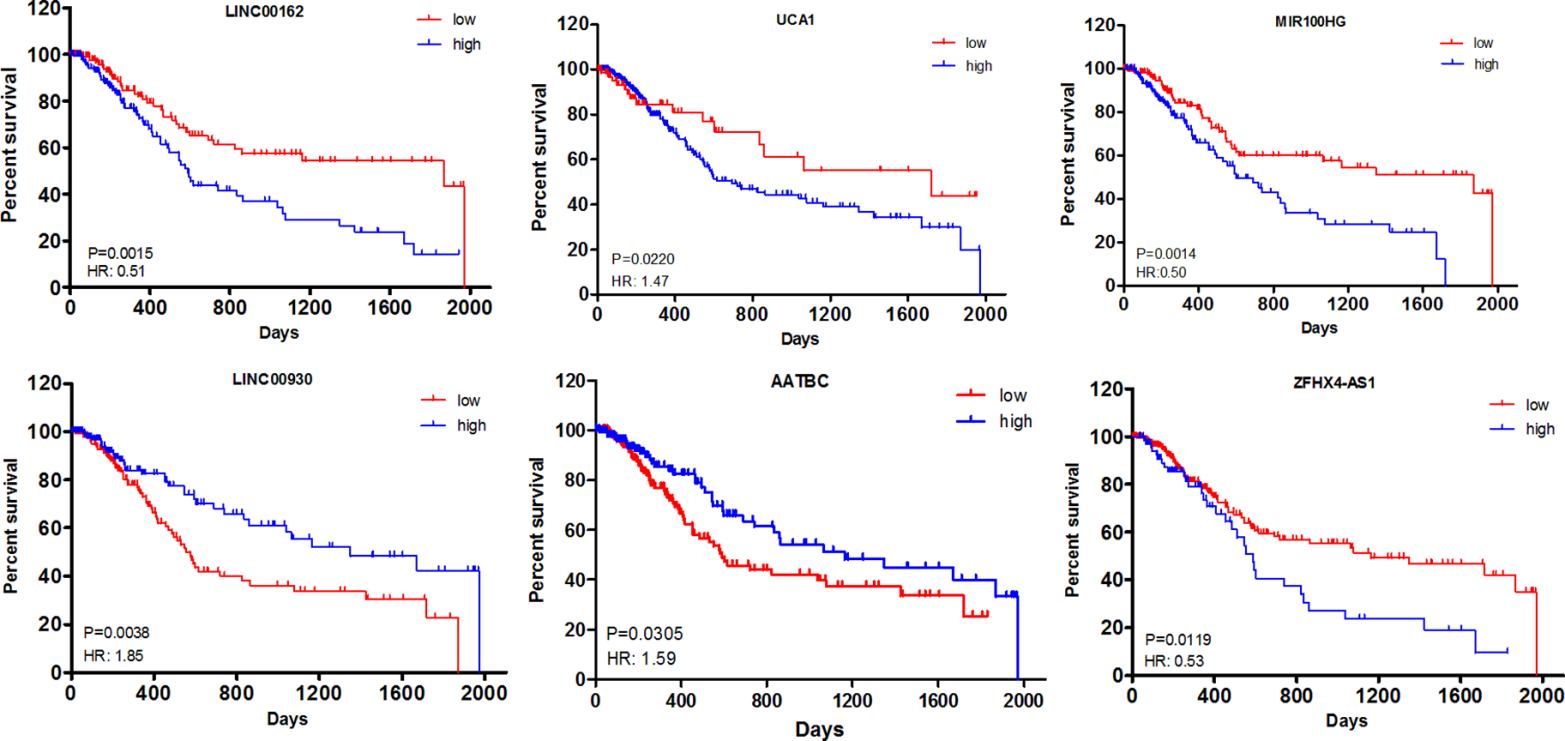
Kaplan–Meier curve analysis of DElncRNAs for the overall survival in muscle-invasive bladder cancer patients Six DElncRNAs were presented (*P* < 0.05), including LINC00162, UCA1, MIR100HG, LINC00930, AATBC and ZFHX4-AS1. HR: hazard ratio (high vs. low). Horizontal axis: overall survival time: days; Vertical axis: survival function

All of the 22 lncRNAs were considered as DElncRNAs that are responsible for the tumorigenesis and/or development of muscle-invasive bladder cancer. Kaplan–Meier curve analysis was then carried out to investigate overall survival for the 22 DElncRNAs in patients with muscle-invasive bladder cancer. As a result, the expression levels of four DElncRNAs, including LINC00162, UCA1, MIR100HG and ZFHX4-AS1, were negatively associated with patient overall survival (*P* < 0.05). On the contrary, two DElncRNAs, LINC00930 and AATBC, prolonged patient survival time as the median survival days were increased from 552 and 590 to 1348 and 1163, respectively (*P* < 0.05) (Figure 3).

### Differentially expressed miRNAs (DEmiRNAs) in muscle-invasive bladder cancer

**Figure 4:**
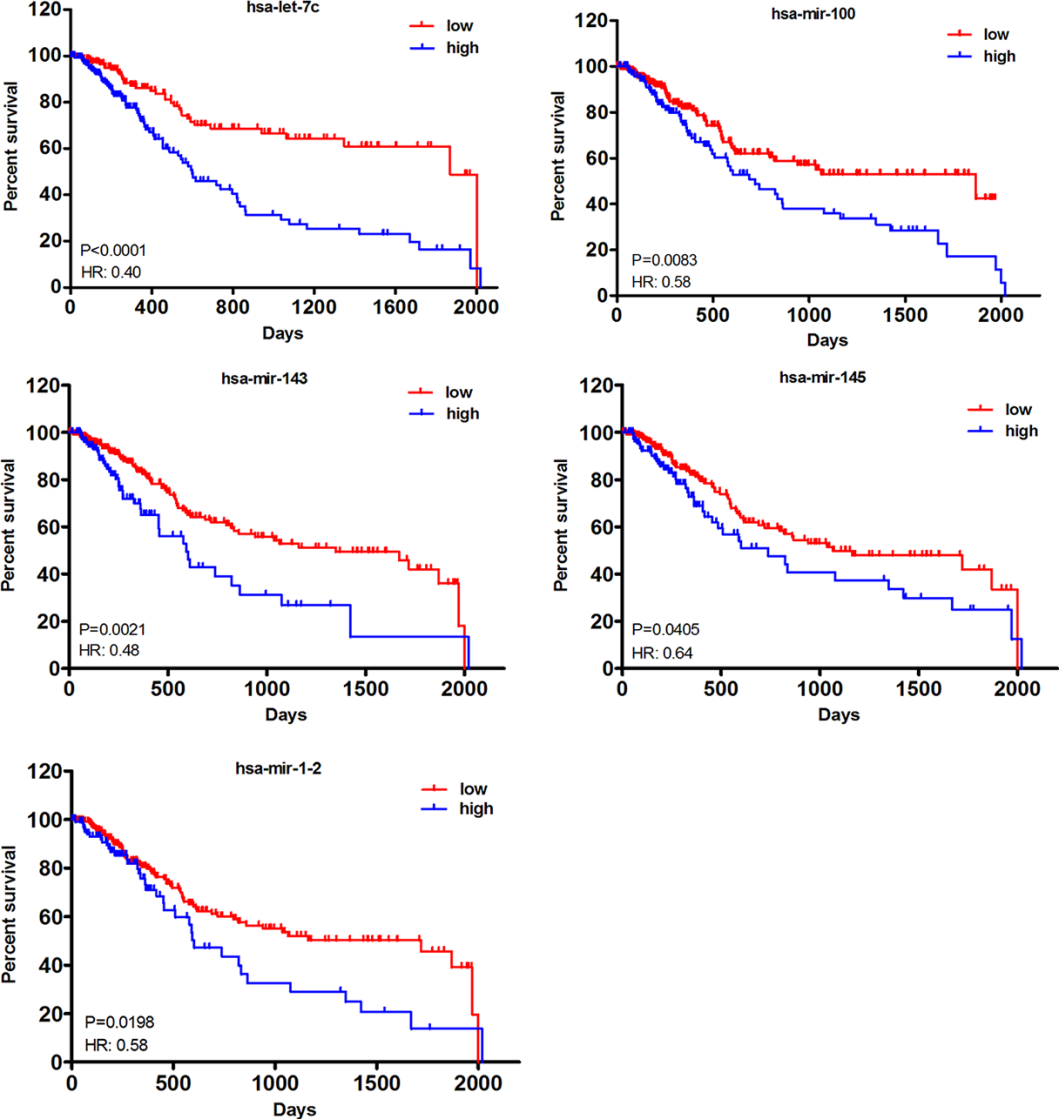
Kaplan–Meier curve analysis of DEmiRNAs for the overall survival in muscle-invasive bladder cancer patients Five DEmiRNAs were presented (*P* < 0.05), including let-7c, mir-100, mir-143, mir-145 and mir-1-2. HR: hazard ratio (high vs. low). Horizontal axis: overall survival time: days; Vertical axis: survival function

To establish an lncRNA-miRNA-mRNA ceRNA network, we also compared miRNA expression profiles in tumor tissues with normal tissues. As shown in Table 3, a total of 11 DEmiRNAs, including 2 up- and 9 down-regulated, were identified. Similar to DElncRNAs, Kaplan–Meier curve analysis was also employed to investigate overall survival for DEmiRNAs in muscle-invasive bladder cancer patients. 5 out of 11 DEmiRNAs, including let-7c, mir-100, mir-143, mir-1-2 and mir-145, were demonstrated to be significantly associated (*P* < 0.05) and all of them exhibited negative effects on patient survival (Figure 4).

**Table 3:** DEmiRNAs in muscle-invasive bladder cancer

Name	Fold Change (T/N)	log2Fold Change(T/N)	*P* Value	FDR
hsa-mir-210	31.74	4.99	2.54E-05	0.004
hsa-mir-183	7.22	2.85	6.03E-05	0.007
hsa-mir-10b	0.34	−1.55	0.00037	0.026
hsa-mir-100	0.25	−2.02	1.47E-05	0.003
hsa-mir-30a	0.24	−2.08	6.49E-09	0.000
hsa-mir-145	0.20	−2.31	3.6E-06	0.001
hsa-let-7c	0.17	−2.53	7.75E-06	0.002
hsa-mir-383	0.09	−3.45	8.97E-05	0.008
hsa-mir-133a	0.08	−3.72	0.000229	0.018
hsa-mir-143	0.07	−3.90	7.27E-05	0.007
hsa-mir-1-2	0.06	−4.01	4.55E-05	0.006

Abbreviations: T: tumor; N: normal.

### ceRNA network in muscle-invasive bladder cancer

**Figure 5:**
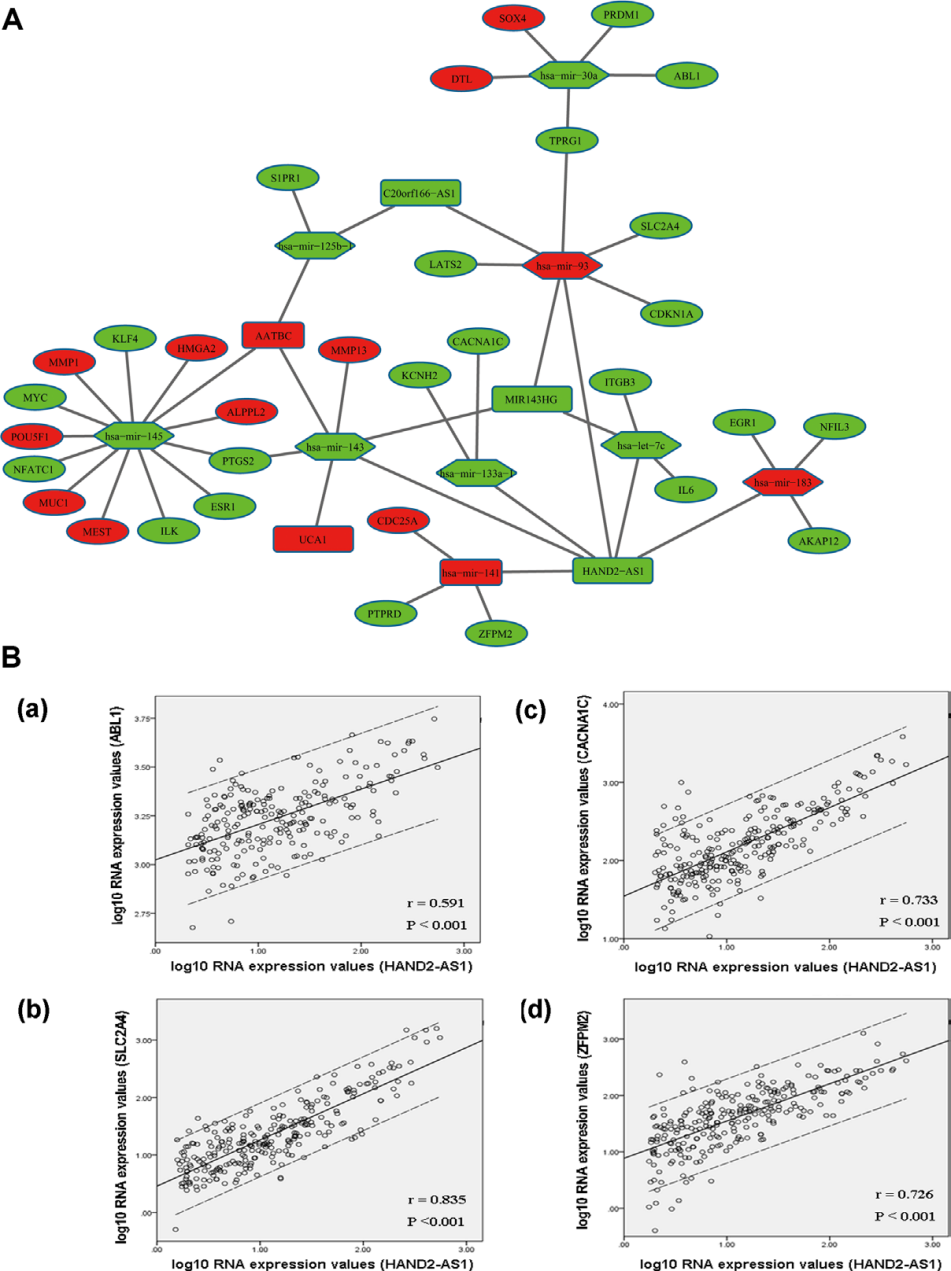
DElncRNA-mediated ceRNA network in muscle-invasive bladder cancer (**A**) Global view of lncRNA-miRNA-mRNA network in muscle-invasive bladder cancer. The nodes highlighted in red indicate increased level of expression, while the nodes highlighted in green indicate decreased level of expression. lncRNAs, miRNAs and protein-coding genes are indicated as round rectangle, hexagon and ellipse, respectively. (**B**) Linear regression of ceRNAs’ expression level. Dashed lines represent 95% confidence interval. (a) HAND2-AS1 vs. ABL1. (b) HAND2-AS1 vs. SLC2A4. (c) HAND2-AS1 vs. CACNA1C. (d) HAND2-AS1 vs. ZFPM2. r: correlation coefficient.

To better understand the function of DElncRNAs in muscle-invasive bladder cancer, we constructed a dysregulated lncRNA-miRNA-mRNA ceRNA network based on above data. In the ceRNA network, we predicted 9 DEmiRNAs could interact with 5 DElncRNAs, according to the results retrieved from miRcode. We searched mRNAs that were targeted by the 9 DEmiRNAs from miRTarBase. mRNAs not included in 1385 DERNAs were excluded. Finally, 32 DERNAs were involved in the ceRNA network. Some of them have been reported to be cancer-associated genes such as CDC25A, CDKN1A, MMP1, MMP13 and SOX4. The network was presented in Figure 5A. We noticed that the DElncRNA HAND2-AS1 interacted with as many as seven DEmiRNAs, including mir-30a, let-7c, mir-133a-1, mir-141, mir-143, mir-183, and mir-93. 21 DERNAs had been experimentally validated to be the targets of these seven DEmiRNAs. We here suppose that HAND2-AS1 may greatly contribute to the pathogenesis of muscle-invasive bladder cancer.

Our constructed ceRNA network showed a possibility that DElncRNAs could indirectly interact with mRNAs in muscle-invasive bladder cancer. To confirm this finding, we performed regression analysis between the expression levels of DElncRNAs and DERNAs that were included in the network. The results revealed a very good or perfect positive correlation between ceRNAs’ expression levels. For example, HAND2-AS1 interacted with SLC2A4, ZFPM2, CACNA1C and ABL1 mediated by mir-93, mir-141, mir-133a-1 and mir-30a, respectively (Figure 5B).

At last, we analyzed the 32 DERNAs that were involved in ceRNA network to reveal signal pathways that indirectly regulated by DElncRNAs. 10 KEGG pathways were significantly enriched in our study (*P* < 0.05) (Table 4). Top 4 KEGG pathways were cancer-related, including ‘MicroRNAs in cancer, Bladder cancer, Cell cycle and Chronic myeloid leukemia’. Other 6 non-cancer related pathways such as ‘Oxytocin signaling pathway and ErbB signaling pathway’ were also enriched.

**Table 4:** KEGG pathways enriched by 32 DERNAs that were involved in the ceRNA network

Pathway ID	Description	Genes
hsa05206	MicroRNAs in cancer	ABL1, CDKN1A, HMGA2, CDC25A, ITGB3, ZFPM2, PTGS2, MYC, SOX4
hsa05219	Bladder cancer	MMP1, CDKN1A, MYC
hsa04110	Cell cycle	ABL1, CDKN1A, CDC25A, MYC
hsa05220	Chronic myeloid leukemia	ABL1, CDKN1A, MYC
hsa04068	FoxO signaling pathway	S1PR1, CDKN1A, IL6, SLC2A4
hsa05410	Hypertrophic cardiomyopathy (HCM)	CACNA1C, ITGB3, IL6
hsa05161	Hepatitis	CDKN1A, NFATC1, IL6, MYC
hsa04012	ErbB signaling pathway	ABL1, CDKN1A, MYC
hsa05020	Prion diseases	IL6, EGR1
hsa04921	Oxytocin signaling pathway	CACNA1C, NFATC1, CDKN1A, PTGS2

## DISCUSSION

A growing body of evidence indicates that lncRNAs play important roles in human cancer. Different studies have shown that aberrant lncRNA expression contribute to the development of different types of cancer [17, 18]. Recently, some efforts have been made to screen aberrantly expressed lncRNAs in bladder cancer and several dysregulated lncRNAs were reported based on microarray data with small sample size [19]. However, studies on identification of the muscle-invasive bladder cancer-specific lncRNAs based on large sample size (*n* = 322) has always been lacking.

Nowadays, lncRNAs are thought to exhibit superior advantage as diagnostic and prognostic biomarkers over protein-coding genes owing to the more precious association between lncRNA expression and tumor status [20]. Several well-studied lncRNAs have been considered as potential targets or powerful predictors in cancers, such as Linc00668, H19 and UCA1 [21–23]. Relevant reports targeting muscle-invasive bladder cancer are rare. Therefore, besides lncRNA expression profiles in muscle-invasive bladder cancer, we also drew our attention to the clinical diagnostic significance of DElncRNAs. In our study, 22 aberrantly expressed lncRNAs were identified and 6 out of them could be considered as prognostic markers for muscle-invasive bladder cancer. In addition, we observed that two DElncRNAs, UCA1 and AATBC, were not only included in ceRNA network but had correlation with overall survival in patient with muscle-invasive bladder cancer, strongly suggesting that the two lncRNAs may serve as key oncogenes as well as prognostic markers in bladder cancer progression. As to UCA1, several reports have revealed its potential in different types of cancer, including breast cancer, ovarian cancer as well as bladder cancer [24–27]. Dysregulated metabolism is one of the classical cancer hallmarks and presents opportunity for cancer diagnostics, prognostics, and therapeutics [28, 29]. In previous report concerning bladder cancer, UCA1 could regulate glucose metabolism in cancer cells through suppressing miR-143 to induce hexokinase 2 [30]. Our analysis confirmed the novel function of UCA1 in bladder cancer and provided another potential mechanism that UCA1 may compete with miR-143 to regulate the expression of MMP13 and PTGS2, both of the coding genes were identified here as dysregulated but lack fully elucidation in bladder cancer. Moreover, it has been demonstrated that knockdown of AATBC could prevent cell proliferation in bladder cancer [31]. The observation is consistent with our identification since patients with high expression level of AATBC exhibited a prolonged survival time. Besides UCA1 and AATBC, MIR100HG, LINC00930, LINC00162 and ZFHX4-AS1 can also serve as significant biomarkers for muscle-invasive bladder cancer. MIR100HG has been considered as oncogene in acute megakaryoblastic leukemia [32]. However, these lncRNAs were firstly determined as prognostic predictors, providing reasonable implications in future clinical practice about targeted therapy of bladder cancer.

Different from miRNAs that have been well studied in various human diseases including bladder cancer [33], only a small number of human lncRNAs have been mechanically and functionally characterized. In our study, we not only identified several specific lncRNAs as well as miRNAs for muscle-invasive bladder cancer in a whole genome wide but also provided a relevant ceRNA network, which will be helpful to further investigation. Many protein coding genes from the newly identified ceRNA network were reported as oncogenes and/or tumor suppressors participating in bladder cancer development and progression, such as CDKN1A, HMGA2 and MYC, which also was considered as promising therapeutic targets for bladder cancer [34–36]. The KEGG pathway analysis results showed that there were four pathways related to cancer. According to the results shown in Table 4 and Supplementary Table S2, we can conclude cell cycle pathway played the most important role in muscle-invasive bladder cancer because it not only appeared to be the most significant cancer-related pathway but also contributed to the regulation of ceRNA network.

Although lncRNAs is rapidly receiving great attentions in the past years, studies focusing on miRNA still remain hot. There is no doubt that finding DEmiRNAs in bladder cancer is necessary for revealing the miRNA-mediated oncogenic pathways. Based on TCGA, a similar work has been done by Dr. Zheng Xu and his colleagues. They screened some potential miRNAs that associated with the progression of muscle-invasive bladder cancer [37]. However, no comparison was made between tumor tissues and normal tissues, nor were miRNAs combined with lncRNAs, coding genes or other types of bioactive molecules. In our study, we identified tumor initiation-related miRNAs in muscle-invasive bladder cancer. Moreover, we revealed how these specific miRNAs interacted with lncRNAs and coding genes because we successfully constructed an lncRNA-miRNA-mRNA ceRNA network. Due to the difference in group of study population, cut-off criteria for the definition of differential expression and method for data procession, no intersection could be found between our study and Dr. Zheng's study. let-7c was the only exception, strongly suggesting its contribution in both tumorigenesis and progression of muscle-invasive bladder cancer.

CeRNAs are widely implicated in many biological processes. It has been confirmed that disorder of ceRNA network can lead to tumorigenesis [38]. Obviously, the ceRNA network we built brings to light an unknown ceRNA regulatory network in muscle-invasive bladder cancer.

## MATERIALS AND METHODS

### Study population

408 bladder cancer patients with RNA sequence data were retrieved from the TCGA data portal. The exclusion criteria were set as follows: 1) for overall survival analysis, patients with follow-up time exceeds 2000 days were excluded (29 cases). and 2) patients without detailed clinicopathological data including age, gender, race, TNM stage, and histological subtype and grade were excluded (57 cases). Overall, a total of 322 bladder cancer patients were included in our study. As the data was obtained from TCGA, further approval by an ethics committee was not required. This study meets the publication guidelines provided by TCGA (http://cancergenome.nih.gov/publications/publicationguidelines).

### RNA sequence data procession

The bladder cancer level 3 RNAseq and miRNASeq data derived from 341 samples, including 322 bladder cancer and 19 normal samples, were downloaded from TCGA. These data were classified into two cohorts. The sequenced data generated from IlluminaHiSeq_RNASeq and IlluminaHiSeq_miRNASeq sequencing platforms were all publicly available data. Each sample consisted of the corresponding RNA-seq and miRNAseq data.

### Analysis of differentially expressed RNAs (DERNAs) and miRNAs (DEmiRNAs)

After tumor sample and normal sample data were combined, the RNA data and miRNA data with no expression were filtered out. Then, the raw count data were processed with DESeq, a Bioconductor package based on R language for differential gene expression analysis using the *nbinomTest* command by following the instructions from the DESeq reference manual [39]. For all the *P*-values, a false discovery rate (FDR) was applied to correct the statistical significance of multiple testing. Genes with absolute fold change (log 2) ≥ 1.5 and the FDR adjusted *P* values < 0.01 were considered as significant.

### Identification of differentially expressed long noncoding RNAs (DElncRNAs)

The GENCODE lncRNA annotation (V22) was used to define lncRNA genes. In our study, noncoding RNAs which are not included in the annotation were discarded. Venn diagram was generated to discover the DElncRNAs from DERNAs.

### Construction of ceRNA network

lncRNA-miRNA interactions were predicted by miRcode (http://www.mircode.org/) [40]. miRNA targeted mRNAs were retrieved from miRTarBase (http://mirtarbase.mbc.nctu.edu.tw/) [41], each miRNA-mRNA pair was experimentally validated by at least two of the following methods including luciferase reporter assay, western blot, qRT-PCR, microarray and ChIP-seq. To further enhance the reliability of the ceRNA network, we filtered the miRNAs and coding genes which were not observed with aberrant expression between tumor tissues and normal tissues. The results were visualized using Cytoscape 3.3.0 [42]. In general gene co-expression network analysis, Pearson's correlation was used as a measure for linear regression.

### Statistical analysis

For overall survival analysis, log-rank test was used to compare significant differences between subgroups using univariate analysis. *P value* less than 0.05 was considered as statistical unless specifically indicated. The statistical analyses were performed using a statistical software package (version 19.0. SPSS, Chicago, IL).

## SUPPLEMENTARY MATERIALS TABLES




