# Supplementary material for: Comprehensive analysis of aberrantly expressed profiles of lncRNAs and miRNAs with associated ceRNA network in muscle-invasive bladder cancer
Source: Oncotarget. 2016 Nov 15;7(52):86174–85. doi: 10.18632/oncotarget.13363 (PMC5349905; doi:10.18632/oncotarget.13363)
Supplement: Supplementary file 1 [file oncotarget-07-86174-s001.pdf]

Comprehensive analysis of aberrantly expressed profiles of lncRNAs and miRNAs with associated ceRNA network in muscle-invasive bladder cancer

Supplementary Materials

Supplementary Table S1: The list of RNAs whose expressions were dysregulated in muscle-invasive bladder cancer. See Supplementary_Table_S1.

Supplementary Table S2: The list of 27 DERNAs that were involved in cell cycle pathway

Gene_Name	Fold_Change[1]	Involve in ceRNA network	Interacted_DEmiRNA	Interacted_DElncRNA
BUB1	3.42	No	N/A	N/A
BUB1B	4.23	No	N/A	N/A
CCNA2	3.50	No	N/A	N/A
CCNB1	3.87	No	N/A	N/A
CCNB2	4.26	No	N/A	N/A
CCNE1	4.93	No	N/A	N/A
CCNE2	4.75	No	N/A	N/A
CDC20	5.41	No	N/A	N/A
CDC25A	3.24	Yes	hsa-mir-141	NBLA00301
CDC45	4.68	No	N/A	N/A
CDC6	4.12	No	N/A	N/A
CDK1	4.98	No	N/A	N/A
CDKN2A	17.84	No	N/A	N/A
E2F1	3.50	No	N/A	N/A
MAD2L1	3.42	No	N/A	N/A
MCM2	3.61	No	N/A	N/A
ORC1	4.62	No	N/A	N/A
ORC6	4.43	No	N/A	N/A
PKMYT1	3.46	No	N/A	N/A
PLK1	4.84	No	N/A	N/A
TTK	4.83	No	N/A	N/A
ABL1	0.31	Yes	hsa-mir-30a	NBLA00301
CCND2	0.28	No	N/A	N/A
CDKN1A	0.33	Yes	hsa-mir-93	C20orf166-AS1
GADD45B	0.15	No	N/A	N/A
MYC	0.31	Yes	hsa-mir-145	AATBC
WEE1	0.29	No	N/A	N/A

1: Tumor vs. Normal. N/A: Not applicable.
